# Tea: Its Effects, Medicinal and Moral

**Published:** 1840-01-01

**Authors:** 


### TEA: ITS EFFECTS, MEDICINAL AND MORAL. By *G. G. Sigmond*, M.D. 8vo. pp. 144. Longman, 1839.

THE opening pages of this elegantly written volume alarmed us a little, lest its accomplished author had been bitten by the TEATOTALO-MANIA of the present day.

"Individually and nationally we are deeply indebted to the tea-plant." "A curious, and not an uninstructive, work might be written upon the singular benefits which have accrued to this country from the preference we have given to the beverage obtained from the tea-plant, above all those that might be derived from the rich treasures of the vegetable kingdom. It would prove that our national importance has been intimately connected with it, and that much of our present greatness, and even the happiness of our social system, springs from this unsuspected source. It would show us that our mighty empire in the East, that our maritime superiority, and that our progressive advancement in the arts and the sciences have materially depended upon it." 3.

We soon discovered, however, that Dr. Sigmond had too much good sense, and too much professional discrimination to be led into this monstrous extreme. Do these tee-totallers imagine that the vine was spread over the most delightful countries in the world by the hand of a beneficent Creator, for no other purpose but to allow its delicious fruit to fall to the earth and rot? Wine is to many constitutions, not only a luxury but a necessary preservative against many diseases which, were it not for the moderate use of this beverage, would assail the body. If they cry out against the pleasant sensations produced by wine, do they mean to say that equally pleasant sensations are not excited by their tea? O, but they tell us, wine produces bad effects in the end. So it does, if taken immoderately:—and so does tea, if taken in excess. Physicians of no mean eminence, even in our own time, (for instance the late Dr. Trotter) have deplored in terms as eloquent as those of Dr. Sigmond, the degeneracy which was rapidly spreading over the present and rising generation, in consequence of the pernicious habits of tea-drinking! The amiable Archdeacon of Bombay has given way to the following Bombay—or rather bombastic absurdity, in his anathema against wine.

"Bacchus, astride of the spirit cask, is the very evil genius of desolation and wretchedness, poverty, disease, and crime; and to have anything to do with his horrid cask, to buy any of it, or to sell any of it, or in any way to lend the respectability of our name in the consumption of it, is downright insanity." 6.

Now if the worthy priest had been seized with the horrible spasms of cholera, while he was inditing the foregoing passage, would he have refused a good dose of laudanum because the menstruum came originally from the cask of Bacchus? Not he.

The sentiments of Dr. Sigmond are very different.

"That dram-drinking is the pernicious source of poverty and sorrow there can be no doubt; but the question may be fairly asked, and duly considered, whether the glass of generous wine, or strengthening beer, is to be totally abandoned, without an examination of the circumstances which may render a moderate enjoyment either prudent or necessary? Must man rush from one extreme to the other? Do not temperature, climate, age, demand some investigation before the denunciation of all fermented liquors be countenanced; and will not even the lover of tea acknowledge his susceptibility of the pleasure and of the utility of his favourite beverage to be heightened by a moderate indulgence in Nature's other gifts? 5.

Upwards of one hundred pages of the volume before us, are occupied with the history, botanical, agricultural, and commercial, of the tea-plant, written in an elegant style, and pregnant with various and useful information. All this we must pass over, in order that we may be able to spare a few pages to that portion of the work which comes more immediately home to our professional avocations and reflections.

After some judicious remarks on diet, and on the necessity for varying it according to the constitution, habits, and avocations of the individual, he comes to the subject of drink, and more especially of tea. He properly observes that, although water is the great beverage of animals and the support of vegetables, it will not suit all constitutions alone. In many countries, districts, and cities, the water is actually bad and unwholesome, not only from mineral, but from animal, or vegetable impregnations. In hot climates the thirst is great; and water alone will not do for all, or even a majority of Europeans transplanted there.

"In almost all warm climates, those who have previously lived in more temperate regions, constantly sip or drink large draughts; but if the first of these habits be acquired, and a bland, slightly bitter fluid such as tea be employed, health will be promoted, and the comfort that it produces will become apparent." 137.

In such cases the beverage of tea or rice-water is preferable to the porter-cup or brandy-shrub. In hot weather, in this country, Dr. S. thinks that tea would be preferable to ice, so commonly had recourse to, for quenching thirst. The following quotation will shew that our author is not yet a disciple of the teatotalo-mania.

"It has been believed, in consequence of some observations made by Mr. Abernethy, that during eating there should be no drinking; and certainly this rule, in some of the diseases of the digestive organs, is important; but it is not to be applied to a state of health. A due admixture of fluid and solid matter is absolutely necessary for healthy action; not large and copious draughts of any fluid whatever, but sufficient to stimulate the salivary glands into their proper secretion, and also enough to propel the already digested mass from the stomach. A gentle stimulus of three or four glasses of wine during the great meal of the day, is the common habit of life of those engaged in occupations which do not demand any very extraordinary exertions, either of body or of mind; and the general state of health, and the longevity of those who do not trespass further upon the limits of moderation, are evident proofs of the propriety of such a system; after the meal, when some little time has elapsed, two or three glasses of Port produce no ill effects. Some individuals only take their wine after the dinner: but this is by no means so serviceable; for the stomach becomes suddenly stimulated, its action is hurried, and the slow and gradual development of heat is exchanged for a sudden excitement, which leaves a greater degree of collapse behind. About two hours after this a diluent may be advantageously taken; then it is that tea imparts a grateful glow of warmth, assists the stomach to unload itself from the digested food, which it gently propels; soon after it has been taken, the languor which is usually attendant upon a full meal disappears, the propensity to slumber so apt to prevail is dissipated, the body feels light, and the mind capable of either gathering fresh information, or of indulging in the recreation which society affords." 109.

Dr. Sigmond condemns the use of strong coffee after dinner, which we have copied from the French. Our strong wines do not require the stimulus of coffee.

"Excitement follows upon its use; watchfulness of a long duration, and a feverish re-action, are amongst its immediate results: its distant ones act upon the extreme capillary vessels of the body, which it seems to constringe, affecting the skin, giving it a peculiar hardness, and it has been affirmed to impart its colour; the sallowness of the skin of the Parisians has been, by more than one medical author, ascribed to it. Many authors have affirmed that paralytic affections, and general debility, follow its use. After dinner, in the form of very strong infusion, the *café noir* is often taken without sugar, sugar-candy, cream, or milk, and is almost an essence of the berry. The individuals to whom it is useful, are those whose breathing is performed with difficulty; they find the greatest relief from drinking strong coffee, and many escape the midnight paroxysm of asthma, by taking their cup about four hours before the usual hour of retiring to rest." 110.

Where individuals can make a good substantial breakfast, tea is better than coffee—and *vice versa*. Tea, without a sufficient quantity of aliment, is apt to influence the nervous system—but so, in fact, is coffee. Cocoa or chocolate is preferable to either, where sufficient bread cannot be taken—the following advice is good.

"Nothing can be more injurious than the habit of taking spirits at breakfast in tea: and this is a very seductive custom, which is followed by persons who complain, that two or three hours after breakfast they feel, without their dram, an uncomfortable sinking at the stomach, a general depression, sometimes palpitation of the heart, and a sense of languor and incapability of moving the limbs, which renders them quite incapable of pursuing their daily avocations." 111.

The rum and milk before getting out of bed is just as bad, though it is recommended by some of the magnates of the profession—whether from personal predilection or general experience, is best known to themselves.

"Tea is more particularly adapted for the ordinary beverage of young women; and the individual who, until the day of her marriage, has never tasted wine, or any fermented liquor, is the one who is most likely to preserve her own health, and to fulfil the great end of her existence, the handing down to posterity a strong and well organised offspring, capable of adding to the improvement and to the welfare of the community." 117.

The following picture of the poisonous effects of green tea on some constitutions, is probably not overcharged—but the more usual effect of this beverage on nervous persons is utter sleeplessness.

"The ordinary effect of green tea taken late at night is incubus or night-mare in its most formidable shape; and many persons, who after a hearty dinner have taken green tea, wake in the midst of the night in a state of the most fearful agitation and excitement: the head is oppressed, a sensation of approaching death is felt, or sometimes the person seems to be dragged from the lowest abyss of darkness back to the world, from which during his paroxysm he had felt gradually to sink. Although none of these symptoms are permanent, and after they have passed away they are forgotten, yet a frequent recurrence of them must lay the foundation of mischief, and ultimately tend to the shortening the duration of life. Many individuals, who have to undergo fatigue, drink quantities of green tea as an antisoporific: certainly it has much power in this way; indeed, it has been successfully employed as an auxiliary to resist the narcotic effects of opium, when it has been too largely taken; but, as the action is that of a sedative upon the heart and arteries, it is injurious, and coffee is much to be preferred, which produces arterial excitement, and thus influences the brain and nervous system, even to the production of exhilaration, which is rarely, if ever, the consequence of the employment of tea." 128.

We must conclude our extracts with the following, which we recommend to the teatotallers, as coming from a physician who is an enthusiastic admirer of tea.

"The vicissitudes of human existence, sometimes in a state of the utmost simplicity, at others of unbounded luxury, demand that aliment suitable to the general wants, as well as to each individual member, should be obtained; that fermented liquors, if injudiciously taken, produce diseased stomachs and livers, consumption, dropsy, madness, is universally acknowledged; and the prudent man, who fears that he may be betrayed into a single excess that may overpower his reason, is perfectly right in shunning the means of mischief. But good wine is a good cordial, a fine stomachic, and taken at its proper season invigorates mind and body, and gives life an additional charm. There can be found no substitutes for the fermented liquors, that can enable man to sustain the mental and bodily labour which the artificial habits of society so constantly demand. Temperance and moderation are virtues essential to our happiness, but a total abstinence from the enjoyments which the bounteous hand of Nature has provided, is as unwise as it is ungrateful. If, on the one hand, disease and sorrow attend the abuse of alcoholic liquors, innocent gaiety, additional strength and power of mind, and an increased capability of encountering the ever-varying agitation of life, are amongst the many good results which spring from a well regulated diet, in which the alcoholic preparations bear their just proportion and adaptation." 131.

From these specimens, selected from only one portion of the work, our readers will be induced to peruse the whole.

